# Cognitive and motor deficits contribute to longer braking time in stroke

**DOI:** 10.1186/s12984-020-00802-2

**Published:** 2021-01-13

**Authors:** Neha Lodha, Prakruti Patel, Joanna M. Shad, Agostina Casamento-Moran, Evangelos A. Christou

**Affiliations:** 1 Department of Health and Exercise Science, Movement Neuroscience and Rehabilitation Laboratory, Colorado State University, Fort Collins, CA 80523 USA; 2 Department of Applied Physiology and Kinesiology, University of Florida, Gainesville, FL USA

**Keywords:** Driving, Visual attention, Divided attention, Selective attention, Motor accuracy, Braking time, Movement

## Abstract

**Background:**

Braking is a critical determinant of safe driving that depends on the integrity of cognitive and motor processes. Following stroke, both cognitive and motor capabilities are impaired to varying degrees. The current study examines the combined impact of cognitive and motor impairments on braking time in chronic stroke.

**Methods:**

Twenty stroke survivors and 20 aged-matched healthy controls performed cognitive, motor, and simulator driving assessments. Cognitive abilities were assessed with processing speed, divided attention, and selective attention. Motor abilities were assessed with maximum voluntary contraction (MVC) and motor accuracy of the paretic ankle. Driving performance was examined with the braking time in a driving simulator and self-reported driving behavior.

**Results:**

Braking time was 16% longer in the stroke group compared with the control group. The self-reported driving behavior in stroke group was correlated with braking time (*r* = − 0.53*, p* = 0.02). The stroke group required significantly longer time for divided and selective attention tasks and showed significant decrease in motor accuracy. Together, selective attention time and motor accuracy contributed to braking time (*R*^*2*^ = 0.40*, p* = 0.01) in stroke survivors.

**Conclusions:**

This study provides novel evidence that decline in selective attention and motor accuracy together contribute to slowed braking in stroke survivors. Driving rehabilitation after stroke may benefit from the assessment and training of attentional and motor skills to improve braking during driving.

## Introduction

Fast and accurate braking is essential to avoid collisions and drive safely [1]. Braking depends on the integrity of cognitive and motor processes [2]. Cognitive skills are required to perceive, extract, and process relevant information from the continuously changing driving environment [3]. Motor skills are necessary to manipulate the gas and brake pedals with accurate and consistent leg movements [4]. However, following stroke, both the cognitive and motor capabilities can be impaired to varying degrees [5, 6]. Consequently, individuals with stroke may experience difficulty in performing driving related tasks such as braking. To date, the influence of cognitive and motor impairments on braking in stroke survivors has not been investigated.

Braking is a critical driving-related task that is associated with collision risk in older adults [7]. Prior research has examined the role of cognitive-motor impairments in overall driving, but not in braking performance following stroke [8–10].Cognitive impairments such as executive dysfunction, attentional deficits, and visuospatial disorientation are a common occurrence after stroke [5, 11, 12]. The decline in visual perception and executive function predict poor driving outcomes and fitness to drive following stroke [13, 14]. Stroke survivors with impaired attention show worse on-road driving skills and are less likely to resume driving [15, 16]. In addition to cognitive impairments, stroke affects motor capabilities in over 70% of survivors [6, 17]. Stroke-related declines in motor functioning, strength, coordination, and range of motion are likely to impact an individual’s ability to accurately operate car controls and influence the decisions for voluntary driving cessation after stroke [18, 19]. To-date, the majority of driving research in stroke has focused on the cognitive factors, and only a few studies have considered motor factors that are arguably essential for driving safely [9, 10]. Given that cognitive and motor impairments occur simultaneously after stroke, it is critical to examine how these deficits impact braking during driving.

The purpose of the current study was to determine the relative contribution of cognitive and motor impairments to braking time in chronic stroke survivors. Prior work has extensively documented that attentional skills are critical for rapid braking response [1, 7]. Therefore, we quantified cognitive function with processing speed, divided attention, and selective attention. Further, decline in ankle muscle strength is shown to be associated with slower braking and unsafe driving in older adults [20, 21]. In addition, we recently demonstrated that decreased ankle position accuracy contributes to impaired braking response in stroke survivors [22]. Therefore, we measured motor function with strength and position accuracy of ankle dorsiflexors and plantarflexors. We measured braking time in a simulated driving environment. Based on previous findings in older adults [20], we hypothesized that motor and cognitive impairments together will be better predictors of braking time than cognitive impairments alone.

## Methods

### Participants

Twenty stroke survivors (64.35 ± 14.83 years) and 20 healthy older adults (67.45 ± 8.39 years) participated in the current study. Inclusion criteria for the stroke participants were as follows: (1) diagnosed with a unilateral cerebrovascular accident at least 6 months prior to testing, (2) current or past drivers, (3) a minimum active range of motion of 15 degrees of ankle dorsiflexion and 5 degrees of active plantarflexion against gravity, and (4) have the ability to understand and follow a three step command (e.g., “Take this piece of paper in your right hand. Fold it in half. Put the paper on the floor.”). Exclusion criteria were (1) presence of any other neurological or musculoskeletal disorder, (2) pain or injury affecting limb movements, (3) spatial neglect, vision, and hearing impairments, (4) psychiatric illness (such as clinical depression or anxiety) or untreated sleep disorder, and (5) history of simulator sickness. The self-reports on these impairments were used to screen the participants. Prior to participation, all individuals read and signed an informed consent approved by the University of Florida’s Institutional Review Board.

### Experimental procedures

The experimental session lasted ~ 3 h. Participants completed clinical, cognitive, motor, and driving assessments.

#### Clinical assessments

We examined the global cognitive status with the Montreal Cognitive Assessment (MoCA)*,* a widely used cognitive screening measure where a lower score indicates impaired cognitive status [23]. In the stroke group, we assessed the severity of motor impairments using lower extremity subsection of the Fugl-Meyer Assessment (FMA), such that a lower score indicates poor motor function. We determined the self-reported driving behavior by current driving status, driving exposure, space, avoidance, and citations [24]. We obtained a self-reported driving score by combining the above factors, with a higher score (maximum 15) indicating superior driving behavior.

#### Cognitive assessments

**Fig. 1:**
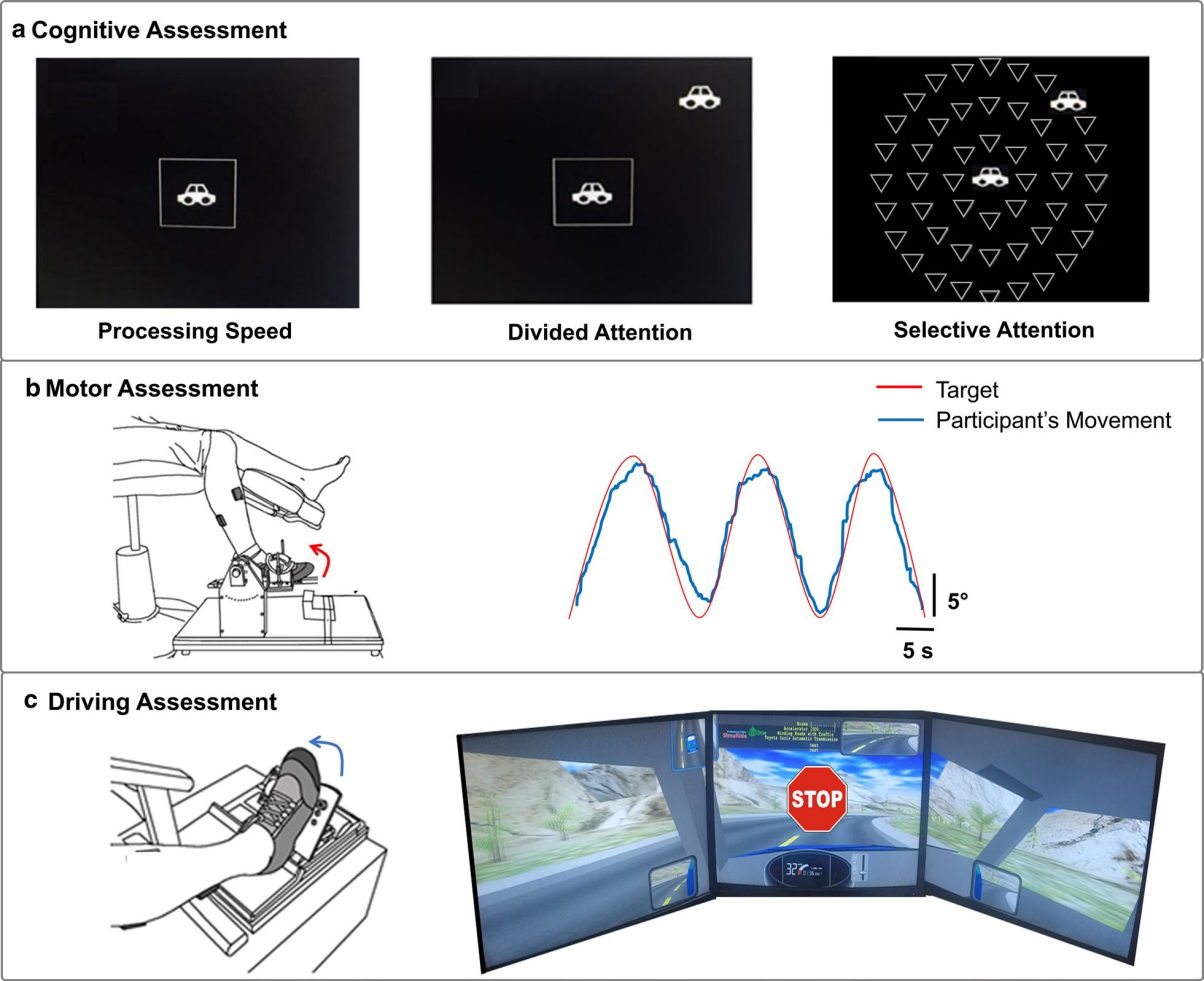
Experimental Procedures: **a** *Cognitive assessments using Useful field of view (UFOV) test*: Participants were asked to identify as fast as possible—a central target (processing speed), a central and a peripheral target simultaneously (divided attention), a central and a peripheral target with distractors (selective attention). **b** *Motor assessment on visuomotor tracking task:* Participants tracked a trajectory (red line) with ankle dorsiflexion and plantarflexion (blue line) movements. **c** *Driving assessment on the simulator:* Participants drove in the center of the lane on a winding road for 3 min. Participants were asked to brake as fast as possible to STOP stimuli displayed at random times

We used the Useful Field of View (UFOV) test to assess processing speed, divided and selective attention [25]. Extensive research suggests that UFOV is a strong predictor of safe driving in older adults [26–28] and stroke survivors[9]. Poor performance on the UFOV test is linked with increased crash risk and decline in driving mobility among older adults [29]. *Experimental set up*: Participants sat in an upright position in front of a 32-inch monitor (Sync Master™ 275t + , Samsung Electronics America, NJ, USA) placed 1.25 m away at eye level. The monitor displayed the UFOV assessment (Fig. 1a).

*UFOV task:* For processing speed, we asked participants to identify a briefly presented stimulus (a car or a truck) in the center of the computer screen. For divided attention, we instructed the participants to identify a central stimulus and simultaneously localize a peripheral stimulus. The selective attention task was identical to the divided attention task except that the peripheral target was embedded within several distractors. *Data measurement and analysis: Processing Speed:* Processing speed measured the time needed to accurately identify a central stimulus. A longer time to correctly identify the central stimuli indicated slower speed of processing. *Divided attention:* Divided attention measured the ability to attend to central and peripheral stimuli simultaneously. A longer time to accurately respond to both stimuli indicated poorer divided attention. *Selective attention:* Selective attention measured the ability to direct attentional processes to two specific stimuli while voluntarily suppressing attention to distractors. A longer time to accurately respond indicated poorer selective attention.

#### Motor assessments

We assessed the maximal force produced with the maximum voluntary contraction (MVC) task and the motor accuracy with the visuomotor tracking task. The motor assessments were performed on the paretic leg in the stroke and the non-dominant leg in the control group.

*Experimental set up* Participants were seated comfortably in an upright position in front of a 32-in. monitor (Sync Master™ 275t + , Samsung Electronics America, NJ, USA) placed 1.25 m away at eye level. The monitor displayed the visual feedback of participants’ performance and the target trajectory (only in visuomotor tracking task). Participants confirmed that they could see the visual display. The hip joint was at ~ 90° flexion and 10° abduction, the knee at ~ 90° flexion, and the ankle in a neutral position. Participants maintained a stable posture and avoided extraneous movements at the hip, knee or trunk. The experimenter monitored the participant’s posture to ascertain compliance.

*MVC task* We asked participants to exert maximum isometric force for 3 s during ankle dorsiflexion and plantarflexion. Each participant completed three to five MVC trials until three MVC trials were within 5% of one another. A 60 s rest period was provided between trials to minimize fatigue. The task order for ankle plantarflexion and dorsiflexion was randomized between participants. *Force measurement and analysis:* A force transducer (Model 41BN, Honeywell, Morristown, NJ, USA) located parallel to the force direction on a customized foot device measured the MVC force. Force signals were sampled at 1000 Hz (NI-DAQ card, Model USB6210, National Instruments, Austin, TX, USA), band-pass filtered from 0.03 to 20 Hz, and amplified by a gain factor of 50 (Bridge-8 world precision instrument Inc., FL, USA). The data were stored on a research workstation for offline analysis. *Strength:* We determined the maximum force for each trial as the average of 10 samples around the peak force. We quantified strength as the highest force obtained among 3–5 MVC trials.

*Visuomotor tracking task* Figure 1b shows the placement of the participant’s foot on an adjustable foot plate secured with Velcro straps to ensure simultaneous movement between the foot plate and participant’s foot. We asked participants to track a sinusoidal target (red line), as accurately as possible using ankle dorsiflexion and plantarflexion movements. Participants received real-time visual feedback of their performance via a blue line that was superimposed on the target. The target frequency was 0.3 Hz. Ankle joint movement ranged from 5° ankle plantarflexion to 15° ankle dorsiflexion. Participants performed 2–3 practice trials and 5 test trials. Each trial lasted ~ 35 s with a 30 s rest period provided between successive trials to minimize fatigue. *Ankle position measurement and analysis*: A low-friction potentiometer (SP22G-5 K, Mouser Electronics, Mansfield, TX, USA) located laterally to the fibular malleolus enabled the measurement of ankle position during the visuomotor tracking task. The ankle position signals were sampled at 1000 Hz (NI-DAQ card, Model USB6210, National Instruments, Austin, TX, USA). Visual presentation of each trial was controlled via a custom routine written in Matlab® (Math Works™ Inc., Natick, Massachusetts, USA). The position signal was band-pass filtered between 0.2 and 0.4 Hz to remove the task-related frequency (sinusoidal target at 0.3 Hz). Data were stored and analyzed offline using a custom routine written in Matlab® program. *Motor Accuracy:* We measured the accuracy of the ankle position as the root mean squared error (RMSE). RMSE quantifies the distance between the target and participant’s position. To account for initial and final position adjustments, we eliminated the first 10 s and the last 5 s of position data from the analysis. We computed the mean RMSE as the average of the 5 trials.

*Driving assessment* We used a commercial driving simulator (AplusB software, Myrtle Beach, South Carolina, 7 USA) to conduct the driving assessment.

*Experimental set-up* Participants sat in a professional driving simulator seat with a gas pedal and a brake pedal. Figure 1c shows placement of the participant’s ankle during the driving task. The simulated driving task was performed with the paretic leg in the stroke and the non-dominant leg in the control group. The simulated driving environment was displayed on three 24-in. computer monitors.

*Simulated driving task* The simulated driving environment included driving a Toyota Yaris on a winding road in clear and sunny weather. We instructed participants to drive in the center of the driving lane at 30 km/h for 3 min. At random times during the driving course, a STOP stimulus would randomly appear. We asked participants to respond to the STOP sign as quickly as possible by releasing the gas pedal and pressing the brake pedal. Prior to testing, participants practiced 2 short driving trials. *Braking Time measurement and analysis:* We measured braking time as the time between the presentation of the STOP stimulus and the application of the brake pedal, averaged over 10 trials. One stroke participant could not complete the simulated driving task and was excluded from the analysis.

### Statistical analysis

We tested the normality of data using the Shapiro–Wilk test. Given that our data were normally distributed, we compared the stroke and control groups using independent samples t-test on (i) cognitive assessments: processing speed, divided attention, and selective attention, (ii) motor assessments: plantarflexion and dorsiflexion strength and accuracy of ankle position, and (iii) braking time. Effect sizes were reported with Cohen’s d. To determine the relationship between cognitive, motor, and driving performance, we performed Pearson’s bivariate correlation. To assess whether motor and cognitive abilities contribute to braking time (criterion variable), we performed a separate hierarchical multiple regression analysis with selective attention as predictor variable in model 1, adding accuracy of ankle position as predictor variable in model 2. The squared multiple correlation coefficient (*R*^*2*^) and the adjusted squared multiple correlation coefficient (adjusted *R*^*2*^) determined the goodness-of-fit of the model. Statistical analysis was conducted with the alpha level set at 0.05 using the IBM SPSS 24.0 (IBM, Armonk, NY).

## Results

### Demographics

The characteristics of study participants are presented in Table 1. The stroke group had a significantly lower MoCA score as compared with the control group (|*t*_38_|= 3.97; *p* = 0.00; *d* = 1.07). The mean FMA score for the stroke group was 27.50 ± 5.53 out of 34. The self-reported driving behavior score trended to be lower in the stroke as compared with the control group, (|*t*_38_|= 1.59; *p* = 0.06;* d* = 0.49).

**Table 1:** Participant characteristics

	Stroke (N = 20)	Control (N = 20)
Age (years)	64.35 ± 14.83	67.45 ± 8.39
Sex (Male/Female), N	8/12	9/11
Height (cm)	170.55 ± 9.88	168.80 ± 7.49
Hemiparetic side (left/right), N	2/18	n/a
Time since stroke (years)	6.10 ± 5.10	n/a
Lesion location		
Cortical	12	n/a
Subcortical	2	n/a
Unknown	6	n/a
MoCA	22.60 ± 4.99	27.30 ± 1.72
FMA	27.50 ± 5.54	n/a
Self-reported driving score	10.25 ± 5.40	12.5 ± 3.26

Self-reported driving score (maximum score 15) included questions about the current driving status, driving exposure, space, avoidance, and citations. All scores are mean ± standard deviation

MoCA, Montreal cognitive assessment (maximum score 30); FMA, Fugl-Meyer motor assessment (maximum score 34); n/a, Not applicable

### Cognitive abilities

**Fig. 2:**
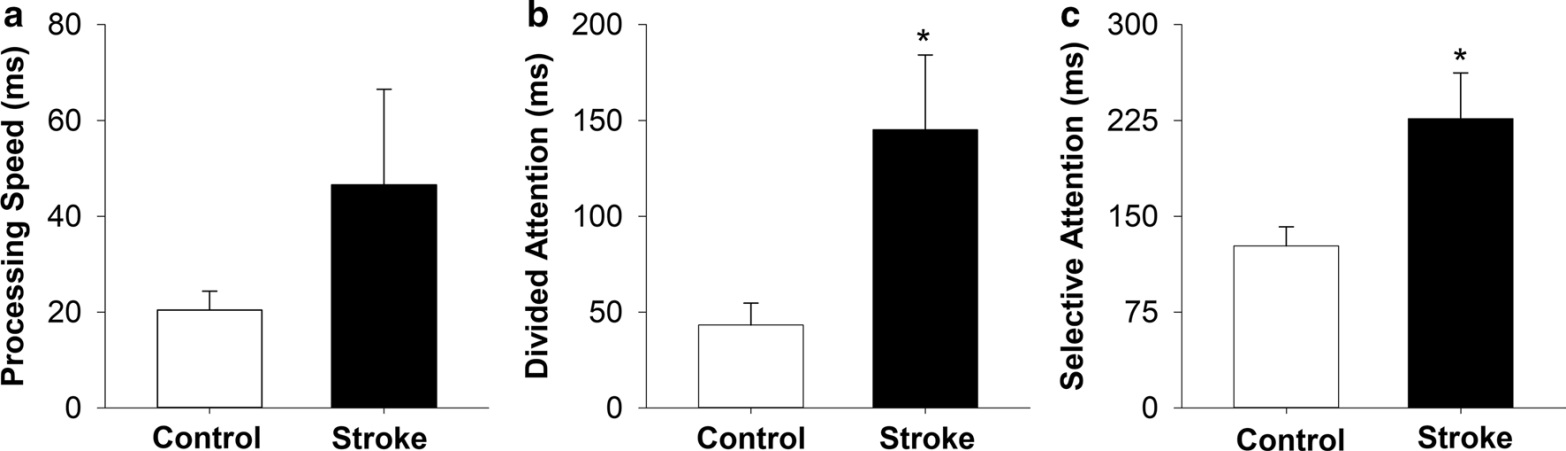
*Cognitive ability:* Cognitive ability was quantified by **a** processing speed, **b** divided attention and **c** selective attention. There was no significant difference between groups on processing speed. The stroke group showed significantly increased time on the divided attention and selective attention tasks compared with the control group. * *p* < 0.05

The stroke group did not differ from the control group on processing speed (|*t*_38_|= -1.28; *p* = 0.21;* d* = -0.40, Fig. 2a). The stroke group showed significantly longer time on the divided attention task (|*t*_38_|= -2.51; *p* = 0.01;* d* = -0.74, Fig. 2b) and selective attention task (|*t*_38_|= -2.57; *p* = 0.01;* d* = -0.76, Fig. 2C) compared with the control group.

### Motor abilities

**Fig. 3:**
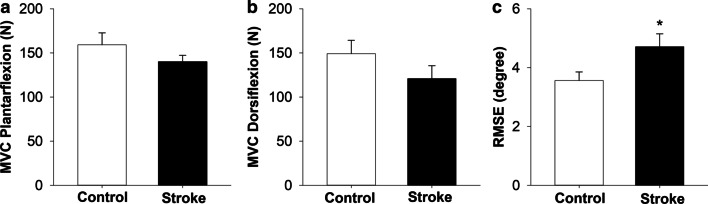
*Motor ability*: Motor ability was quantified by **a** plantarflexion MVC **b** dorsiflexion MVC, and **c** RMSE (motor accuracy) during ankle plantarflexion and dorsiflexion of the paretic limb. The stroke and the control group did not differ significantly on ankle dorsiflexion and plantarflexion MVC (strength). The stroke group showed significant increase in RMSE (reduced motor accuracy) as compared with the control group. * *p* < 0.05

The two groups did not differ significantly on plantarflexion MVC (|*t*_38_|= 1.24; *p* = 0.22;* d* = 0.39, Fig. 3a) or dorsiflexion MVC (|*t*_38_|= 1.34; *p* = 0.18;* d* = 0.42, Fig. 3b). We excluded two stroke participants who could not complete the MVC task from this analysis. The stroke group had significantly higher RMSE of the paretic ankle position compared with the non-dominant ankle position in the control group (|*t*_38_|= -2.17; *p* = 0.03;* d* = -0.66, Fig. 3c).

### Braking performance

**Fig. 4:**
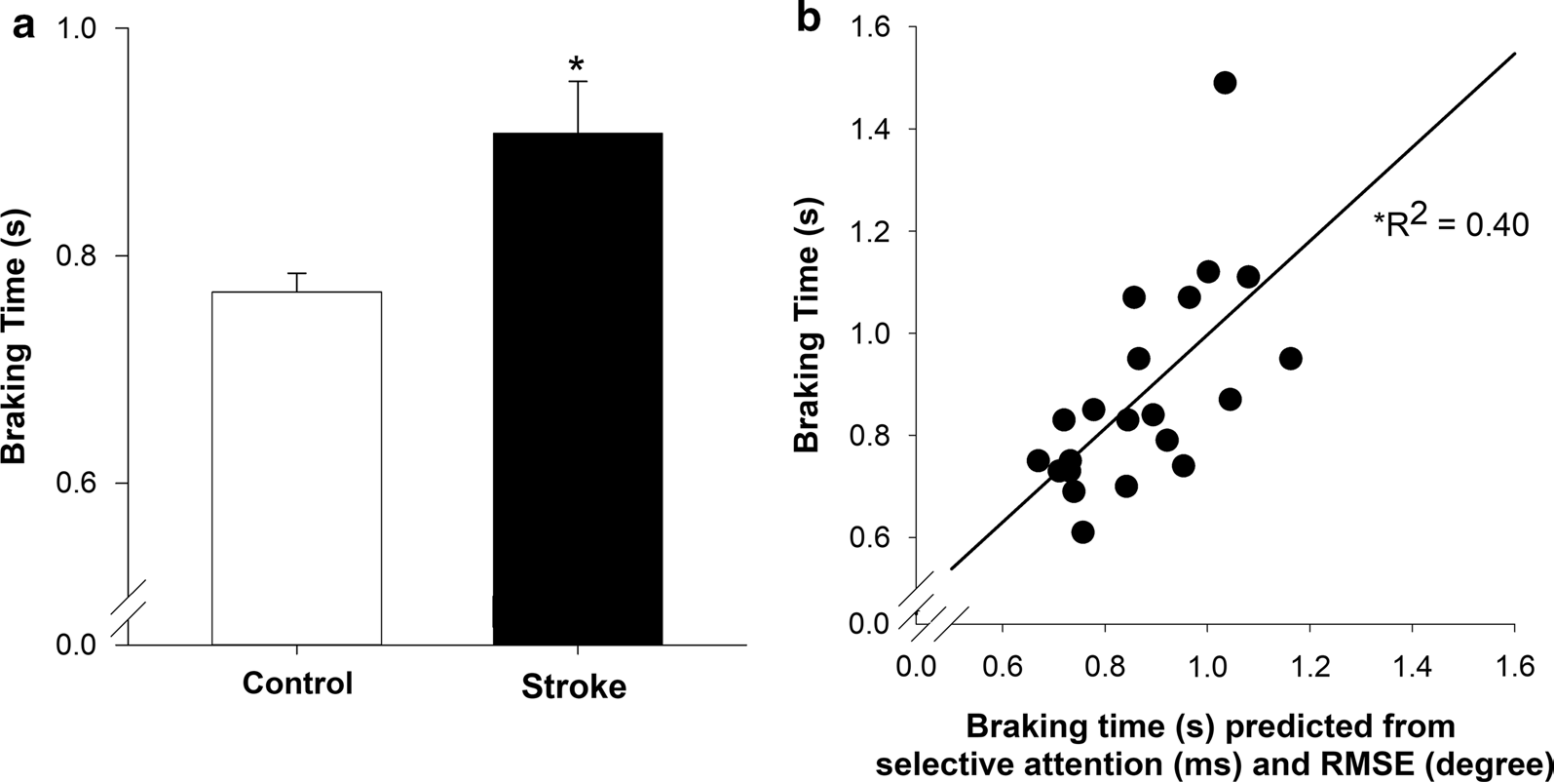
Simulated driving performance **a** stroke group demonstrated significant increase in braking time compared with the control group and **b** selective attention and RMSE (motor accuracy) significantly contributed to braking time in the stroke group. * *p* < 0.05

The braking time was significantly longer in the stroke group compared with the control group, (|*t*_37_|= − 2.45; *p* = 0.02;* d* = − 0.74, Fig. 4a).

### Relation between cognitive, motor, and driving performance in stroke

Table 2 shows the correlation results with braking time. Braking time significantly correlated with selective attention (*r* = 0.44, *p* = 0.03) and RMSE (*r* = 0.62, *p* = 0.005). Further, braking time was significantly correlated with self-reported driving behavior score (*r* = − 0.53, *p* = 0.02). Finally, selective attention was correlated to divided attention (*r* = 0.88, *p* = 0.00).

**Table 2:** Pearson correlations between braking time, cognitive, motor function

	Selective attention		Divided attention		Processing speed		Dorsiflexion MVC		Plantarflexion MVC		RMSE	
	*r*	*p*	*r*	*p*	*r*	*p*	*r*	*p*	*r*	*p*	*r*	*p*
Braking time	*0.44*	*0.03*	0.34	0.07	0.12	0.31	− 0.005	0.98	− 0.05	0.85	*0.62*	*0.005*

Significant correlations are shown in italics

To determine the contribution of cognitive and motor impairments to the braking time in the stroke group, we ran a hierarchical multiple regression analysis with selective attention and accuracy of ankle position as predictor variables. We determined selective attention and RMSE as our cognitive and motor predictor variables because of their significant correlation with braking time. Our results indicate that selective attention and RMSE of the paretic ankle contributed to braking time (*R*^*2*^ = 0.40*, adjusted R*^*2*^ = 0.33*, p* = 0.01*,* Fig. 4b). Prediction model 1 showed that selective attention alone accounted for 19.09% of the variance in braking time, however, did not reach the significance level, (*F*_(1, 17)_ = 4.01, *p* = 0.06). Prediction model 2 showed that selective attention and RMSE were significant predictors of braking time, (*F*_(2, 16)_ = 5.38, *p* = 0.01) and, together they accounted for 40.20% of the total variance in braking time. The *R*^*2*^ change for Model 2 was significant, (*F*_(1, 16)_ = 5.66, *p* = 0.03). Finally, increased RMSE (part *r* = 0.46) and increased selective attention (part *r* = 0.13*)* contributed to longer braking time in chronic stroke survivors.

## Discussion

The current study determined the contribution of cognitive and motor impairments to braking time in chronic stroke survivors. Overall, braking time was 16% longer in the stroke group compared with the control group. The impairments in selective attention and motor accuracy contributed to the longer braking time in stroke survivors. For the first time in the stroke literature, we provide novel evidence that impairments in both cognitive and motor domains are significant contributors to braking performance in chronic stroke – a key determinant of safe driving.

### Stroke increases the time required to apply brakes during simulated driving

Braking is a key component of driving function. Deficits in braking can increase the distance that a car travels following hazard detection, thus elevating the chances of collision and road injury. A national report suggested that braking-related problems accounted for 22 percent of vehicle crashes nationwide [30]. Depending on the car speed and driver’s expectation of events, a few milliseconds of delay in braking can significantly increase the chances of a traffic accident [2]. In the current study, we assessed braking time in a simulated driving task that required attending to the driving environment, recognizing the STOP stimulus and planning the braking action (cognitive component) as well as rapidly releasing the gas pedal, moving the foot to brake pedal and applying braking force (motor component). Our findings suggest that the time to apply brakes was significantly increased in the stroke compared with the control group, suggesting that longer time is required to slow or stop a car in an unexpected traffic incident post-stroke. Further, the longer braking time was associated with poor self-reported driving behavior in stroke survivors. Thus, braking time may serve as a surrogate measure of overall driving behavior in stroke.

Stroke impairs the ability to execute the braking response, a task that relies on adequate attention to rapidly recognize relevant traffic events and skilled foot movements to apply accurate braking forces [10]. We focused on processing speed, selective, and divided attention as these cognitive functions relate with the crash risk in older adults [26, 31]. Our results show that relative to the controls, the stroke group had slower speed of divided and selective attention, skills that are necessary for focusing on task relevant information to meet the demands of rapidly changing traffic conditions while driving. Clearly, stroke impairs the ability to filter out unwanted information while focusing on relevant features of a task (selective attention). Further, stroke affects an individual’s ability to rapidly shift attention between two tasks (divided attention). Our results are in line with previous findings showing stroke-related decrease in attentional skills [25, 32]. Overall, chronic stroke survivors demonstrated persistent attentional deficits that can interfere with braking performance during driving.

While the link between cognitive dysfunction and poor driving outcomes after stroke has been documented extensively [9, 33], the relationship between motor dysfunction and driving in stroke is not well-studied. In our study, we demonstrate that stroke reduced the accuracy of ankle movements. The motor control of the ankle is important for accurate placement of the foot on the brake and gas pedals, applying precise brake forces, and modulating the car speed. Simulated and on-road driving assessments show that stroke survivors are more likely to exceed speed limits, have poor speed adaptations, and have difficulty maintaining safe trailing distance from the lead vehicle according to the driving speed [33, 34]. Lack of adequate motor control in stroke can contribute to such driving errors. For example, stroke survivors with impaired upper-limb motor control demonstrate poor steering and increased lane deviation during simulated driving [35]. Similarly, age-related deterioration in ankle motor control was associated with poor reactive driving [4]. Interestingly, despite persistent impairments in motor accuracy of the paretic ankle, there was no difference in ankle muscle strength between the stroke and control groups. Adequate muscle strength may be important for producing sufficient ankle force to press the gas and brake pedals, especially during extended driving duration. However, in our study, we did not find ankle strength to be significant predictor of braking time. One possible reason could be that our simulated driving test lasted for only a few minutes and required quick but moderate force levels to change the brake pedal position. Thus, the stroke survivors with reasonable strength recovery had acquired adequate strength to drive a short duration and generate the required brake forces. Our findings support that even after sufficient strength recovery, braking performance in stroke is impacted by reduced movement precision.

### Cognitive and motor impairments collectively influence braking time in stroke.

The most interesting finding of our study is that the braking time in the stroke group was contributed by cognitive and motor impairments collectively, rather than cognitive impairments alone. Together, selective attention and motor accuracy contributed to 40% of the variance in braking time. Introducing motor accuracy in Model 2, contributed to an additional 20.1% variance in the braking time. It seems intuitive that a complex task such as braking may involve multiple functional domains i.e., cognitive and motor processes. However, empirical evidence regarding this proposition is lacking in stroke-driving literature. The current study provides novel insights into the importance of attention and movement precision in braking performance post-stroke. These results are in line with previous studies. For example, Aslaksen et al., 2013 showed that on-road driving performance in traumatic brain injury and stroke was predicted by visuomotor information processing speed and motor dexterity [36]. Similarly, Alonso et al., 2016 reported that braking time in older adults was associated with postural control, muscle strength, and global cognitive function [20]. Finally, it is possible that our assessments of cognitive and motor function are interdependent since the measurement of cognitive ability relies on motor dexterity [37] and the measurement of motor accuracy can depend on visual information processing [38, 39]. Despite this limitation, we selected these assessments as they are the most used measures of cognitive and motor function in the literature [15, 25, 40, 41]. Overall, our results illustrate the importance of assessing both cognitive and motor domains to understand the factors that may potentially limit critical driving-related tasks such as braking performance after stroke.

### Considerations and clinical implications

Braking indicates the time required to slow or stop a car in response to an unexpected sign or object. Thus, braking is a relatively small yet key component of overall driving ability. We tested braking in the simulated driving environment. Understanding the relative contribution of cognitive-motor function to on-road driving performance in stroke requires future investigation. A key strength of our study is that our results yielded moderate effect sizes for the influence of stroke on braking time, selective attention, and motor accuracy. Further, selective attention and motor accuracy explained significant variance in braking performance. Together these results strengthen our proposition that cognitive and motor impairments are significant contributors to braking performance in chronic stroke.

Even though motor impairments are fairly apparent after stroke, the visual and cognitive factors are typically more emphasized in driving assessments following stroke [42, 43]. This is not highly surprising given the availability of car adaptations such as steering knobs, pedal extensions etc., to circumvent motor impairments that interfere with safe driving. We argue that adequate use of car modifications relies on residual motor capabilities and relearning a motor task that was previously performed by another limb or was performed in a different way by the same limb. Our results indicating the combined influence of motor and cognitive impairments to braking provide a compelling reason for assessing and rehabilitating both motor and cognitive deficits for favorable driving outcomes post-stroke.

A potential limitation of the study is the strict inclusion and exclusion criteria. Most participants in the current study had relatively mild motor impairments (FMA score = 27.50 ± 5.54) and demonstrated voluntary movement control to complete the simulated driving task. Future studies should test braking in individuals with severe motor impairments, provided they can operate the car controls. Further, 18 out of 20 participants with stroke had a left hemisphere lesion. In the current study, we examined braking with the paretic right leg which was reported as the leg used for driving by all, except two of our participants. These factors limit the generalizability of the results. To expand the ecological validity of these findings, future studies should include equal number of individuals with right/left hemisphere lesion and investigate braking performance with the typical driving leg whether non-paretic or paretic. Our findings have potential clinical implications on driving assessment in stroke survivors and illustrate the importance of assessing both cognitive and motor domains to understand and treat the factors that limit critical driving-related tasks such as braking.

## Conclusion

In summary, our study provides novel evidence that persistent impairments in motor and cognitive functions contribute to braking deficits in stroke survivors. Slower braking following stroke can increase the distance that a car travels before it stops or slows down in response to a hazard detection. Stroke survivors required longer time to selectively focus or shift attention between tasks and demonstrated impaired movement precision despite the absence of decline in muscle strength. Most importantly, braking time in stroke survivors was contributed by selective attention and the accuracy of ankle movements, rather than selective attention alone. Therefore, clinicians should consider assessment and rehabilitation of both motor and cognitive impairments for improving braking performance for safe driving post-stroke.

## Data Availability

Data and materials can be provided upon request.
